# Decreasing Incidence and Prevalence of Dementia Among Octogenarians: A Population-Based Study on 3 Cohorts Born 30 Years Apart

**DOI:** 10.1093/gerona/glad071

**Published:** 2023-02-27

**Authors:** Hanna Wetterberg, Jenna Najar, Therese Rydberg Sterner, Lina Rydén, Hanna Falk Erhag, Simona Sacuiu, Silke Kern, Anna Zettergren, Ingmar Skoog

**Affiliations:** Neuropsychiatric Epidemiology Unit, Department of Psychiatry and Neurochemistry, Institute of Neuroscience and Physiology, Sahlgrenska Academy, Centre for Ageing and Health (AgeCap) at the University of Gothenburg, Sweden; Neuropsychiatric Epidemiology Unit, Department of Psychiatry and Neurochemistry, Institute of Neuroscience and Physiology, Sahlgrenska Academy, Centre for Ageing and Health (AgeCap) at the University of Gothenburg, Sweden; Region Västra Götaland, Sahlgrenska University Hospital, Psychiatry, Cognition and Old Age Psychiatry Clinic, Gothenburg, Sweden; Neuropsychiatric Epidemiology Unit, Department of Psychiatry and Neurochemistry, Institute of Neuroscience and Physiology, Sahlgrenska Academy, Centre for Ageing and Health (AgeCap) at the University of Gothenburg, Sweden; Aging Research Center, Department of Neurobiology, Care Sciences and Society, Karolinska Institutet and Stockholm University, Stockholm, Sweden; Neuropsychiatric Epidemiology Unit, Department of Psychiatry and Neurochemistry, Institute of Neuroscience and Physiology, Sahlgrenska Academy, Centre for Ageing and Health (AgeCap) at the University of Gothenburg, Sweden; Neuropsychiatric Epidemiology Unit, Department of Psychiatry and Neurochemistry, Institute of Neuroscience and Physiology, Sahlgrenska Academy, Centre for Ageing and Health (AgeCap) at the University of Gothenburg, Sweden; Neuropsychiatric Epidemiology Unit, Department of Psychiatry and Neurochemistry, Institute of Neuroscience and Physiology, Sahlgrenska Academy, Centre for Ageing and Health (AgeCap) at the University of Gothenburg, Sweden; Region Västra Götaland, Sahlgrenska University Hospital, Psychiatry, Cognition and Old Age Psychiatry Clinic, Gothenburg, Sweden; Neuropsychiatric Epidemiology Unit, Department of Psychiatry and Neurochemistry, Institute of Neuroscience and Physiology, Sahlgrenska Academy, Centre for Ageing and Health (AgeCap) at the University of Gothenburg, Sweden; Region Västra Götaland, Sahlgrenska University Hospital, Psychiatry, Cognition and Old Age Psychiatry Clinic, Gothenburg, Sweden; Neuropsychiatric Epidemiology Unit, Department of Psychiatry and Neurochemistry, Institute of Neuroscience and Physiology, Sahlgrenska Academy, Centre for Ageing and Health (AgeCap) at the University of Gothenburg, Sweden; Neuropsychiatric Epidemiology Unit, Department of Psychiatry and Neurochemistry, Institute of Neuroscience and Physiology, Sahlgrenska Academy, Centre for Ageing and Health (AgeCap) at the University of Gothenburg, Sweden; Region Västra Götaland, Sahlgrenska University Hospital, Psychiatry, Cognition and Old Age Psychiatry Clinic, Gothenburg, Sweden

**Keywords:** Epidemiology, Cognition, Community-based, Time trends

## Abstract

**Background:**

Recent studies suggest a decline in the age-specific incidence and prevalence of dementia. However, results are mixed regarding trends among octogenarians. We investigated time trends in the prevalence and incidence of dementia in 3 population-based cohorts of 85–90-year olds. We also examined if there were different time trends for men and women.

**Methods:**

We examined population-based birth cohorts within the Gothenburg H70 Birth Cohort Studies born 1901–02, 1923–24, and 1930, at ages 85 (*N* = 1481) and 88 (*N* = 840) years. The first 2 cohorts were also examined at age 90 (*N* = 450). The incidence was examined in 1 109 individuals free from dementia at baseline using information from the examination at age 88 or register data. All 3 cohorts were examined with identical methods.

**Results:**

The prevalence of dementia decreased from 29.8% in 1986–87 to 21.5% in 2008–10 and 24.5% in 2015–16 among 85-year olds, and from 41.9% in 1989–90 to 28.0% in 2011–12 to 21.7% in 2018–19 among 88-year olds, and from 41.5% in 1991–92 to 37.2% in 2013–14 among 90-year olds. The decline was most accentuated among women. The incidence of dementia per 1 000 risk-years from ages 85 to 89 declined from 48.8 among those born 1901–02 to 37.9 in those born 1923–24 to 22.5 among those born 1930.

**Conclusions:**

The prevalence and incidence of dementia decreased substantially over 3 decades among octogenarians. This might slow down the projected increase in cases of dementia expected by the increasing number of octogenarians during the following decades.

Dementia is one of the major causes of disability and dependency among older adults (1). Due to increased life expectancy, the number of people with dementia is projected to increase globally from 57 million in 2019 to 152 million by 2050 (2). Most dementia cases occur in the population above age 80 (3).

The prevalence of dementia reflects the burden of disease on society, whereas the incidence of dementia gives information on the risk for disease. Therefore, it is important to report both prevalence and incidence data when analyzing time trends in dementia. For the planning of dementia health policies and costs of health services, it is essential to have robust and up-to-date estimates of the age-specific prevalence and incidence of dementia and to know whether these estimates have changed over time (4).

Several population-based studies suggest a decline in the prevalence and incidence of dementia in Western countries (5–16), potentially slowing down the predicted rise in dementia cases. A declining dementia incidence in the population is partly suggested to be a result of a later age of onset (8,17), which could imply an increasing incidence in the higher age groups. Previous studies on time trends in the epidemiology of dementia in octogenarians are, however, mixed with reports of increasing incidence (18), no change in incidence (6), or a decreasing age-specific prevalence (16) or incidence (15) of dementia. Some studies report that women have had a more pronounced decline in the incidence and prevalence of dementia in later-born birth cohorts (8,10), others show a decrease among men (9), and some have reported no sex differences (5,6). This is important to consider as women are reported to have a higher prevalence and incidence of dementia after age 85 (19).

To investigate time trends in dementia incidence and prevalence, rigorous population-based studies using the same criteria and examination methods over time are necessary. Another important issue when investigating dementia incidence is how to handle losses to follow-up. Using register data is one way, although there is a risk of low sensitivity to detect dementia in registers, with decreasing sensitivity with increasing age (20). When using register data in studies of time trends, it is vital to know about changes in sensitivity and specificity to detect the outcome.

Based on reported societal changes and risk factors for dementia, we hypothesized that the prevalence and incidence of dementia have decreased in more recently born birth cohorts. As higher education is associated with a lower risk of dementia (21), and women have had an increasing access to education during the past century which has led to increased cognitive function in women (22), we also hypothesized that this decrease was more accentuated among women. We used data from 3 population-based cohorts, born 1901–02, 1923–1924, and 1930, who were examined at ages 85, 88, and 90 years to investigate time trends in the prevalence and incidence of dementia.

We also examined if there were different time trends for men and women. In addition, we investigated the sensitivity and specificity of dementia diagnoses in the National Inpatient Register (IPR; Swedish: Patientregistret) and the cause of death register (CDREG; Swedish: Dödsorsaksregistret).

## Method

### Study Population

Three cohorts of 85-year olds from the Gothenburg H70 Birth Cohort Studies living in Gothenburg, Sweden, and born 1901–02, 1923–24, and 1930 were examined in 1986–87 (*N* = 494; response rate 64.5%), 2008–10 (*N* = 571; response rate 60.5%) and 2015–17 (*N* = 416; response rate 61.9%; see Supplementary Figure 1 for details regarding sampling procedure).

Follow-ups were conducted at age 88 (1989–90, *N* = 260; 2011–12, *N* = 322; and 2018–19, *N* = 258) and age 90 (1991–92, *N* = 200 and 2013–14, *N* = 250). The cohort born in 1930 could not be examined at age 90 due to the coronavirus disease-2019 (COVID-19) pandemic. All 3 cohorts were systematically obtained from the Swedish Population Register based on birth dates to yield a representative sample. Both individuals living in private households and institutions were included, and the only exclusion criteria applied was an inability to speak Swedish. The selected individuals received an invitation letter to participate in a health examination, which either took place at the Old Age Psychiatry Clinic at Sahlgrenska University Hospital or in the participant’s home (private household or institution). Only individuals who participated in the examination at age 85 were invited for examinations at ages 88 and 90 in the cohorts born 1901–02 and 1923–24. In the 1930 cohort, the sampling included all individuals born on the specified birth dates at age 88.

Participants and nonparticipants did not differ regarding sex in any cohorts at age 85. In cohort 1901–02, there were no differences between participants and nonparticipants in mortality rate up to age 88, but mortality was lower among participants than in nonparticipants in the cohorts 1923–24 and 1930.

### Examinations

The examinations were as similar as possible between the 3 cohorts to ensure comparability, as described previously (16,23). The semi-structured neuropsychiatric examinations, performed by a psychiatrist in 1986–87 and by experienced psychiatric research nurses in all the other study waves, included assessments of psychiatric symptoms, signs of dementia, tests of mental functioning (eg, memory, proverbs, language, visuospatial and executive abilities, apraxia, and agnosia), the Mini-Mental State Examination (24) and the Alzheimer’s disease assessment scale cognitive subscale—ADAS-Cog (25).

The examinations in 1986–87 were performed by the principal investigator (I.S.), who also trained and supervised the psychiatric research nurses who performed all later examinations. Dual ratings by nurses or psychiatrists tested interrater reliability for signs and symptoms used to diagnose dementia. Interrater agreement was 89.4%–100.0% (kappa values 0.74–1.00).

Semi-structured interviews with close informants were performed at all examinations and comprised questions about changes in behavior and intellectual function (eg, changes in personality, memory, intellectual ability, language, visuospatial function, psychiatric symptoms, and activities of daily living).

### Diagnosis of Dementia

The diagnostic procedures were identical at all examinations and have been described in detail previously (23). First, a diagnosis of dementia was made from the psychiatric examination and the close informant interview separately using an algorithm based on the Diagnostic and Statistical Manual of Mental Disorders, 3rd ed., revised (DSM-III-R) criteria (26), except that impairment in short-term *or* long-term memory was sufficient to fulfill the requirements of memory impairment. The participants were diagnosed with dementia if they fulfilled the criteria in both the psychiatric examination and close informant interview or if they fulfilled the criteria in 1 source and had support for a diagnosis from the other. To ensure comparability, 2 senior psychiatrists independently reviewed the algorithmic outputs in all 3 cohorts, followed by a consensus conference. Output failures that resulted in diagnosis disagreement implied further review of the research case record by the same psychiatrists until a consensus was reached.

For the study of incidence, we also used the IPR and information from the CDREG for those who were lost to follow up with codes from the Swedish version of the International Classification of Diseases (ICD) 8 (290, 293.0, 239.1), ICD 9 (290, 249B, 331A, 331B, 331C, and 331X) and ICD 10 (F00, F01, F02, F03.9, G30, G31.1, and G31.8A). Both primary and secondary diagnoses were included. The IPR was established in 1964 and has had full coverage in the region since 1973 (27). It is mandatory for both private and publicly funded physicians to report data to the IPR. The CDREG had full coverage in the nation since 1961 and contains information on all deceased in Sweden and the cause of death (27).

### Date of Death

Information on the date of death was available from the national registration administered by Statistics Sweden.

### Ethical Approval and Informed Consent

Informed consent was obtained from all participants, and in cases of severe dementia, the examination was carried out with consent from a close relative. The Regional Ethical Review Board approved the study, and all methods were performed in accordance with the Helsinki Declaration.

### Statistical Methods

Logistic regression was used to compare the cohorts pairwise regarding the prevalence of dementia at ages 85, 88, and 90. The COVID-19 pandemic precluded the examination of cohort 1930 at age 90, and the dementia prevalence at age 90 was only compared between cohorts 1901–02 and 1923–24. Model 1 was adjusted for sex. To examine the effect of education on birth cohort differences, we added educational level (more vs less than mandatory education) to Model 2. To explore time trends by sex, we also ran Model 1 and Model 2 for men and women separately. We also compared the differences in the prevalence of dementia between men and women within the cohorts. The study-specific estimates were standardized by sex and education to the 2021 Swedish census data (28).

The person-year incidence rates were calculated as follows: All dementia-free individuals who participated at baseline contributed with person-years until death or dementia diagnosis (either according to the examination at age 88, or collected from the registers at age 89 based on registry data availability until December 31, 2019). We did not include data from the examination at age 90 to be able to compare all 3 cohorts regarding incidence because cohort 1930 was not examined at this age. The time of dementia onset was considered to have occurred midway between the first examination and the follow-up at age 88, or the date when dementia diagnosis was first recorded according to the registry. The incidence rate was then calculated by dividing the incident cases by the sum of person-years, and the 95% confidence intervals (CIs) for proportions were calculated for the estimated rates. The 4-year cumulative incidence from age 85 was modeled with Poisson regression as a function of cohort and sex, with a natural log person-years follow-up offset term. Model 1 was adjusted for sex in the total group, and the interaction term Cohort × Sex was used to derive the incidence rates by sex. In Model 2 we added educational level.

When analyzing sensitivity and specificity for dementia diagnoses in the IPR or CDREG, all participants at ages 85 and 88 were included, including all cases of dementia. Dementia diagnoses from the examinations at ages 85 and 88 were used as a gold standard. Sensitivity refers to the proportion of dementia diagnoses in the examinations that were also identified by the IPR or CDREG up to age 89, whereas specificity refers to the proportion of nondementia cases in the examinations that were noncases also in the IPR or CDREG. The 95% CIs for proportions were calculated for the estimated proportions.

We used SAS (Statistical Analysis Software 9.4, SAS Institute Inc, Cary, North Carolina, USA), SPSS, version 28, and STATA, version 15, for statistical analyses.

## Results

The sample characteristics are shown in Table 1.

**Table 1.** Characteristics in the 3 Cohorts at Ages 85, 88, and 90

Characteristics	Cohort		
	1901–02	1923–24	1930
Participants at age 85, (*n*)	494	571	416
Age (mean ± *SD*)	85.5 ± 0.1	85.9 ± 0	86.0 ± 0.2
Sex (women), (%) (*n*)	71.1 (351)	62.9 (359)	60.3 (251)
More than basic education, (%) (*n*/*N*)	24.9 (113/454)	56.0 (311/555)	53.0 (212/400)
sMMSE (mean ± *SD*) (*n*)	23.8 ± 7.7 (491)	25.1 ± 6.5 (555)	25.3 ± 7.1 (410)
Married, (%) (*n*/*N*)	23.9 (117/490)	35.5 (195/550)	39.0 (161/413)
Participants at age 88, (*n*)	260	322	254*
Age (mean ± *SD*)	88.2 ± 0.2	88.6 ± 0.2	88.6 ± 0.2
Sex (women), (%) (*n*)	73.8 (192)	63.7 (205)	63.4 (161)
More than basic education, (%) (*n*/*N*)	26.9 (65/242)	63.0 (199/316)	52.4 (130/248)
sMMSE (mean ± *SD*) (*n*)	20.2 ± 9.1 (258)	25.1 ± 6.8 (322)	25.9 ± 7.2 (251)
Married, (%) (*n*/*N*)	19.0 (49/258)	28.6 (91/318)	30.9 (68/220)
Participants at age 90, (*n*)	200	250	N/A
Age (mean ± *SD*)	90.2 ± 0.2	90.5 ± 0.4	N/A
Sex (women), (%) (*n*)	71.5 (143)	64.0 (160)	N/A
More than basic education, (%) (*n*/*N*)	29.8 (57/191)	63.4 (154/243)	N/A
sMMSE (mean ± *SD*) (*n*)	21.6 ± 8.6 (196)	23.6 ± 8.6 (249)	N/A
Married, (%) (*n*/*N*)	16.5 (33/200)	28.9 (72/249)	N/A

*Notes*: N/A = not applicable; *N* = number; *SD* = standard deviation; sMMSE = standardized Mini-Mental State Examination (58). Cohort 1930 was not examined at age 90 due to the COVID-19 pandemic.

*Four participants excluded due to missing information on dementia diagnosis.

### Prevalence of Dementia

**Figure 1.**
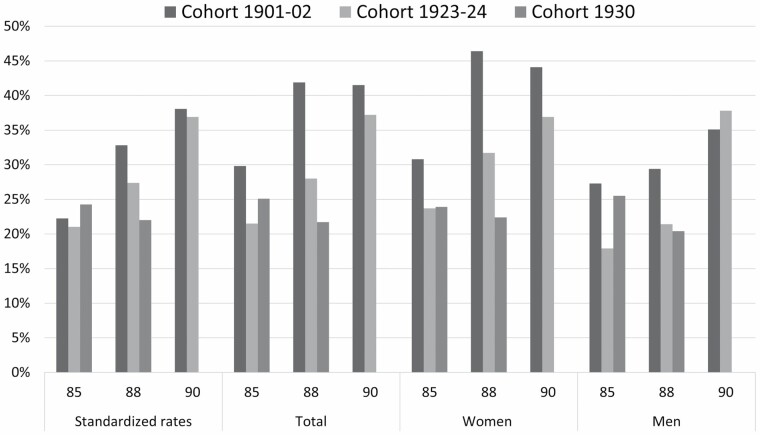
Dementia prevalence at ages 85, 88, and 90 in cohorts 1901–02, 1923–24, and 1930. Standardized rates based on 2021 Swedish census data.

Crude and standardized prevalence rates of dementia in each birth cohort at ages 85, 88, and 90 are shown in Figure 1 (for exact estimates, see Supplementary Table 1), and the results from the logistic regressions using Model 1 and Model 2 are shown in Table 2. The prevalence of dementia at age 85 in cohorts 1901–02 and 1923–24 has been described previously (16).

**Table 2.** Regression Models Comparing Prevalent Dementia Between Birth Cohorts

	Total				Women			Men	
	*n*	OR (95% CI)	*p* Value	*n*	OR (95% CI)	*p* Value	*n*	OR (95% CI)	*p* Value
Age 85									
Model 1									
1901–01 (reference)									
1923–24	1 065	0.66 (0.50–0.87)	.004	710	0.70 (0.50–0.97)	.034	355	0.58 (0.35–0.97)	.037
1930	910	0.77 (0.57–1.04)	.086	602	0.71 (0.49–1.02)	.065	308	0.91 (0.55–1.51)	.718
1930 vs 1923–24	987	1.19 (0.88–1.60)	.260	610	1.01 (0.69–1.48)	.948	377	1.56 (0.95–2.57)	.077
Model 2									
1901–01 (reference)									
1923–24	1 009	0.95 (0.69–1.29)	.729	669	1.04 (0.71–1.50)	.854	340	0.77 (0.44–1.35)	.361
1930	859	1.04 (0.75–1.45)	.797	566	0.96 (0.65–1.44)	.855	293	1.22 (0.69–2.15)	.497
1930 vs 1923–24	960	1.17 (0.86–1.59)	.305	589	1.00 (0.68–1.48)	.992	371	1.52 (0.92–2.51)	.099
Age 88									
Model 1									
1901–01 (reference)									
1923–24	582	0.57 (0.40–0.80)	.001	397	0.54 (0.36–0.81)	.003	185	0.65 (0.33–1.29)	.220
1930	514	0.40 (0.27–0.59)	<.001	353	0.33 (0.21–0.53)	<.001	161	0.62 (0.30–1.27)	.191
1930 vs 1923–24	576	0.71 (0.48–1.05)	.085	366	0.62 (0.39–0.996)	.048	210	0.94 (0.48–1.85)	.868
Model 2									
1901–01 (reference)									
1923–24	558	0.72 (0.49–1.07)	.101	376	0.78 (0.50–1.23)	.288	182	0.62 (0.30–1.29)	.199
1930	490	0.52 (0.34–0.78)	.002	333	0.46 (0.28–0.75)	.002	157	0.69 (0.32–1.47)	.331
1930 vs 1923–24	564	0.72 (0.49–1.07)	.101	355	0.62 (0.38–1.01)	.057	209	0.95 (0.48–1.85)	.872
Age 90									
Model 1									
1901–01 (reference)									
1923–24	450	0.84 (0.58–1.24)	.382	303	0.74 (0.47–1.18)	.204	147	1.12 (0.56–2.24)	.742
Model 2									
1901–01 (reference)									
1923–24	434	0.92 (0.61–1.38)	.675	289	0.84 (0.51–1.39)	.497	145	1.11 (0.53–2.33)	.776

*Notes*: CI = confidence interval; OR = odds ratio.

At age 85, the prevalence of dementia decreased from 29.8% in cohort 1901–02 to 21.5% in cohort 1923–24 (odds ratio [OR] = 0.66; 95% CI: 0.50–0.87, *p* = .004), whereas the prevalence of 24.5% in cohort 1930 did not differ significantly from cohort 1901–02 (OR = 0.77; 95% CI: 0.57–1.04, *p* = .086), or from cohort 1923–24 (OR = 1.19; 95% CI: 0.88–1.60, *p* = .260). At age 88, the prevalence of dementia decreased from 41.9% in cohort 1901–02 to 28.0% in cohort 1923–24 (OR = 0.57; 95% CI: 0.40–0.80, *p* = .001) to 21.7% in cohort 1930 (OR = 0.40; 95% CI: 0.27–0.59, *p* < .001). Cohort 1923–24 and 1930 did not differ significantly from each other (OR = 0.71; 95% CI: 0.48–1.05, *p* = .085). At age 90, the prevalence of dementia was 41.5% in cohort 1901–02 and 37.2% in cohort 1923–24 (OR = 0.84; 95% CI: 0.58–1.24, *p* = .382).

We then examined birth cohort differences by adjusting the models by education. At age 85, the difference in prevalence between cohorts 1901–02 and 1923–24 disappeared in the total sample (OR = 0.95; 95% CI: 0.69–1.29, *p* = .729). At age 88, the differences in prevalence between cohorts 1923–24 and 1901–02 disappeared (OR = 0.72; 95% CI: 0.49–1.07, *p* = .101), but remained between cohorts 1930 and 1901–02 (OR = 0.52; 95% CI: 0.34–0.78, *p* = .002).

When stratifying by sex, the prevalence of dementia among women at age 85 decreased from 30.8% in cohort 1901–02 to 23.7% in cohort 1923–24 (OR = 0.70; 95% CI: 0.50–0.97, *p* = .034), whereas the prevalence of 23.9% in cohort 1930 did not significantly differ from cohort 1901–02 (OR = 0.71; 95% CI: 0.49–1.02, *p* = .065), or from cohort 1923–24 (OR = 1.01; 95% CI: 0.69–1.48, *p* = .948). Among men, the prevalence of dementia at age 85 decreased from 27.3% in cohort 1901–02 to 17.9% in cohort 1923–24 (OR = 0.58; 95% CI: 0.35–0.97, *p* = .037), whereas the prevalence of 25.5% in cohort 1930 did not differ significantly from cohort 1901–02 (OR = 0.91; 95% CI: 0.55–1.51, *p* = .718), or from cohort 1923–24 (OR = 1.56; 95% CI: 0.95–2.57, *p* = .077). At age 88, the prevalence of dementia among women decreased from 46.4% in cohort 1901–02 to 31.7% in cohort 1923–24 (OR = 0.54; 95% CI: 0.36–0.81, *p* = .003) and 22.4% in cohort 1930 (OR = 0.33; 95% CI: 0.21–0.53 *p* < .001), and was lower in cohort 1930 compared to 1923–24 (OR = 0.62; 95% CI: 0.39–0.996, *p* = .048). Among men, the prevalence of dementia was 29.4% in cohort 1901–02, 21.4% in cohort 1923–24 (OR = 0.65; 95% CI: 0.33–1.29, *p* = .220), and 20.4% in cohort 1930 (OR = 0.62; 95% CI: 0.30–1.27, *p* = .191) and did not differ significantly between cohorts 1930 and 1923–24 (OR = 0.94; 95% CI: 0.48–1.85, *p* = .868). At age 90, the prevalence of dementia among women was 44.1% in cohort 1901–02 and 36.9% in cohort 1923–24 (OR = 0.74; 95%  CI: 0.47–1.18, *p* = .204). Among men, the prevalence of dementia was 35.1% in cohort 1901–02, and 37.8% in cohort 1923–24 (OR = 1.12; 95% CI: 0.56–2.24, *p* = .742).

We then examined the effect of education on birth cohort differences stratified by sex. At age 85, the difference between cohort 1923–24 and 1901–02 among both men and women disappeared (women, OR = 1.04; 95% CI: 0.71–1.50, *p* = .854, and men OR = 0.77; 95% CI: 0.44–1.35, *p* = .361). At age 88, the difference in prevalence in women between cohorts 1923–24 and 1901–02 disappeared (OR = 0.78; 95% CI: 0.50–1.23, *p* = .288), as well as between cohort 1930 and 1923–24 (OR = 0.62; 95% CI: 0.38–1.01, *p* = .057), but remained between cohorts 1930 and 1901–02 (OR = 0.46; 95% CI: 0.28–0.75, *p* = .002; Table 2).

The sex and education standardized prevalence figures show that with a similar distribution, the prevalence at age 85 would be similar across all 3 cohorts, decline at age 88, and remain similar at age 90.

We then compared the prevalence of dementia between men and women within each of the 3 cohorts (Supplementary Table 2). At ages 85 and 90, there were no differences between men and women within the cohorts, but at age 88, women had, compared to men, a higher prevalence of dementia in cohorts 1901–02 and 1923–24, but not in cohort 1930 (Supplementary Table 2).

### Incidence of Dementia Between Ages 85 and 89 Years

**Figure 2.**
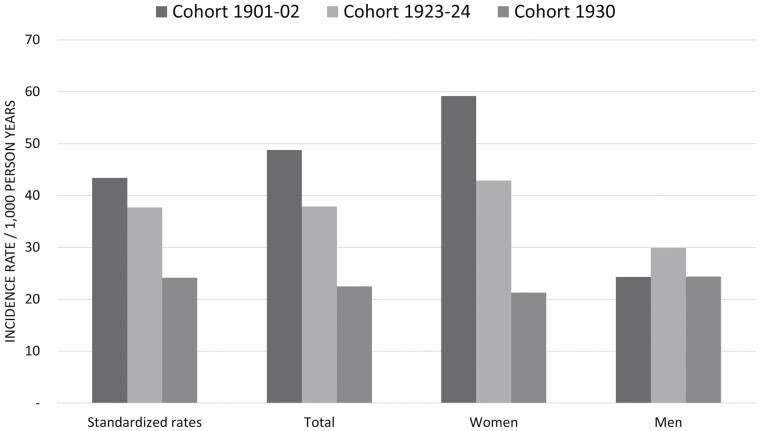
Four-year dementia incidence rates in cohorts 1901–02, 1923–24, and 1930. Standardized rates based on 2021 Swedish census data.

The 4-year incidence rate of dementia per 1 000 person-years from age 85 to 89 years for each birth cohort is presented in Figure 2 (for exact estimates, see Supplementary Table 3), and the incidence rate ratios from the Poisson regression using Model 1 and Model 2 are shown in Table 3. Between ages 85 and 89 years, the incidence of dementia was 49/1 000 person-years in cohort 1901–02, 38/1 000 person-years in cohort 1923–24, and 23/1 000 person-years in cohort 1930. This represents a decline between cohort 1930 and 1901–02 (incident rate ration [IRR] = 0.48; 95% CI: 0.28–0.82, *p* = .007), but not between cohorts 1923–24 and 1901–02 (IRR = 0.80; 95% CI: 0.54–1.21, *p* = .296), nor between 1930 and 1923–24 (IRR = 0.59; 95% CI: 0.35–1.01, *p* = .056). When adjusting for education, the difference in incidence remained between cohorts 1930 and 1901–02 (IRR = 0.48; 95% CI: 0.28–0.83, *p* = .008).

**Table 3.** Poisson Regression Models Predicting Incidence of Dementia Between Age 85 and 89

	Total			Women			Men		
	*n*	(IRR, 95% CI)	*p* Value	*n*	(IRR, 95% CI)	*p* Value	*n*	(IRR, 95% CI)	*p* Value
Model 1									
1901–01 (reference)									
1923–24	795	0.80 (0.54––1.21)	.296	517	0.72 (0.46–1.15)	.175	278	1.23 (0.50–3.05)	.653
1930	661	0.48 (0.28–0.82)	.007	434	0.36 (0.18–0.70)	.003	227	1.00 (0.36–2.77)	.995
1930 vs 1923–24	762	0.59 (0.35–1.01)	.056	465	0.50 (0.25–0.99)	.045	297	0.81 (0.34–1.94)	.644
Model 2									
1901–01 (reference)									
1923–24	778	0.79 (0.52–1.21)	.287	506	0.72 (0.44–1.16)	.173	272	1.23 (0.49–3.07)	.659
1930	648	0.48 (0.28–0.83)	.008	426	0.37 (0.19–0.72)	.004	222	1.00 (0.36–2.77)	.994
1930 vs 1923–24	744	0.61 (0.36–1.04)	.069	452	0.51 (0.26–1.02)	.057	292	0.81 (0.34–1.93)	.636

*Notes*: IRR = incident rate ratios; CI = confidence intervals; *N* = number included in analysis. Model 1, adjusted for sex; Model 2, adjusted for sex and education.

The incidence of dementia among women was 59/1 000 person-years in cohort 1901–02, 43/1 000 years in cohort 1923–24, and 21/1 000 person-years in cohort 1930. This represented a decline among women between cohorts 1930 and 1901–02 (IRR = 0.36; 95% CI: 0.18–0.70, *p* = .003), and between cohorts 1930 and 1923–24 (IRR = 0.50; 95% CI: 0.25–0.99, *p* = .045), but not between cohorts 1923–24 and 1901–02 (IRR = 0.72; 95% CI: 0.46–1.15, *p* = .175). When adjusting for education, the difference in incidence remained between cohorts 1930 and 1901–02 (IRR = 0.37; 95% CI: 0.19–0.72, *p* = .004) but not between cohorts 1930 and 1923–24 (IRR = 0.51; 95% CI: 0.26–1.02, *p* = .057).

In men, displaying no differences in incidence rates between cohorts, we found rates of 24/1 000 person-years in cohort 1901–02, 30/1 000 person-years in cohort 1923–24, and 24/ 000 person-years in cohort 1930 (cohort 1923–24 vs 1901–02, IRR = 1.23; 95% CI: 0.50–3.05, *p* = .653, cohort 1930 vs 1901–02, IRR = 1.00; 95% CI: 0.36–2.77, *p* = .995, and cohort 1930 vs 1923–24, IRR = 0.81; 95% CI: 0.34–1.94, *p* = .644).

The sex and education standardized incidence shows that with a similar distribution, the estimated incidence would be lower in both cohorts 1923–24 and 1930 compared to cohort 1901–02.

We then compared the incidence of dementia between men and women within each of the 3 cohorts (Supplementary Table 4). The incidence rate of dementia was higher among women compared to men in cohort 1901–02, whereas there were no sex differences in cohorts 1923–24 and 1930.

### Sensitivity and Specificity of the IPR or CDREG to Detect Dementia

The total number of dementia cases in the 3 cohorts during the study period was 492 (Supplementary Table 5). The IPR or CDREG identified 216 of these cases and yielding a sensitivity of 43.9% (95% CI: 39.5–48.4). The total number of noncases was 1 031, and the IPR or CDREG identified 1 014 of these as noncases, yielding a specificity of 98.4% (95% CI: 97.4–99.0). The sensitivity and specificity were similar for all 3 cohorts. See Supplementary Table 6 for estimates for the IPR only.

## Discussion

We found a decrease in the prevalence and incidence of dementia over 3 decades between the 1980s and the 2010s in a population-based study of octogenarians from Gothenburg, Sweden, born from 1901 to 1930. Higher education partly explained the declining prevalence in the later-born cohorts. The most accentuated decline was observed among 88-year olds and women.

Our finding of a decreased incidence of dementia in a population from Sweden is supported by several other population-based studies from Western countries conducted in mixed ages (6,8–10,15,29,30). In 2020, the ACC consortium published data from 11 European and North American cohorts and reported a consistent decrease in dementia incidence since 1998 (30). These results are further supported by studies based on registered data, such as a reported decline in hospital dementia incidence in Sweden between 2011 and 2016 (31) and in Denmark between 2010 and 2015 (32). However, reports vary on whether this decrease continues into very old ages, and few studies have reported specifically on the age group above 85 years. In line with our results is a study from Sweden showing a decrease in the incidence of dementia up to age 89 years from 108/1 000 person-years to 70/1 000 person-years, comparing a cohort examined in 2000 with a cohort examined in the late 80s (15), and the MRC Cognitive Function and Ageing study (MRC CFAS) study from the United Kingdom, suggested a decline in dementia incidence from 62/1 000 person-years to 49/1 000 between 1989–94 and 2008–11 in individuals aged 85 years and older (9). Our IRs, which decreased from 49/1 000 to 23/1 000 person-years between 1986–87 and 2015–16, are lower than in the other studies, maybe due to more examination points during follow-up in the other studies. Some studies are in contrast to our results showing no change in incidence, such as the Framingham study. Their results showed no change in incidence among those aged 80 years and older between 1977 and 2008, despite a declining incidence in the age group 60–79 years (8). Similar patterns were reported from the Rotterdam study comparing cohorts 2000–07 (6) and a study from Indianapolis comparing the early 1990s and the early 2000s (29).

Also, the prevalence of dementia decreased between the first and middle cohorts at age 85, but the prevalence in the most recent cohort did not differ significantly from the other cohorts. However, the estimates are in the same direction and the smaller sample size in this cohort might suggest a power issue. At age 88, the prevalence decreased continuously between cohorts from 42% to 28% to 22%, and at age 90, the trend of a decline continued, however, not statistically significant. A decline in prevalence, which reflects the societal burden of disease, is important in an age group where most dementias occur. We have recently reported that mortality decreases to the same extent in those with and without dementia but with continued higher mortality in those with dementia (33), similar to findings from the Health and Retirement Study (34). The finding that the relation in mortality between those with and without dementia remains stable between cohorts, suggests that the decrease in prevalence to a larger extent is linked to a reduced incidence of dementia. A decreasing prevalence of dementia has also been reported in several other Western countries in populations aged above 65 (11,12,14), with some exceptions (35,36). Few studies have reported on time trends in dementia prevalence after age 80 or 85 years. A study from Finland using self-reported dementia diagnoses in nonagenarians showed a declining prevalence from 2007 to 2018, from 47% to 41% (37). A Spanish study comparing the prevalence of dementia between 1988–89 and 1994–96 reported no change after age 85, with prevalence of 16% and 18% (38), the MRC-CFAS study reported prevalence of 24% and 16% in the late 80s and 2010s in populations aged 85–89, a decline which was not statistically significant (11). A Swedish study reported a stable prevalence of around 20% among 85–89-year olds and closer to 40% among 90-year olds (35), similar to a rural Swedish study reporting prevalence of 22% and 17% in a population aged 78+ in 1995 and 2001 (12). These estimates are overall in line with our findings, with the exception of lower prevalence in the Spanish and the rural Swedish. The lower figures in the rural Swedish study might be explained by the inclusion of younger age groups, and the Spanish study used a 2-phased diagnostic procedure.

One explanation for disparate results in a change in incidence and prevalence among octogenarians could be the time period for the final examination, which was 2015 in our study and 2013 in MRC-CFAS (9), whereas studies reporting no decline had their final examination baselines before 2000–01 (6,29,38). The decline in the oldest age group may thus have occurred recently, mainly in those born after the 1920s, which is supported by findings from the Einstein Aging Study Cohort (39). Other explanations could be differences in response rates, especially in the higher age groups, and small subgroups among octogenarians, giving rise to low statistical power. It could also be due to factors varying by geographical area, such as sociodemographic disparities, culture, race and ethnicity, and political and legal systems interacting and differently affecting the risk of dementia (40).

It has been suggested that a decline in the prevalence and incidence of dementia among later-born cohorts results from improvement in risk or protective factors for dementia over time (14,40). Factors such as education, lifestyle, and medical treatment have drastically changed during the last century in connection with the development of the welfare state in Western countries (41). We have previously reported a decline in several risk factors for dementia, for example, sleep problems (42), neuroticism and depression (43), obesity and overweight, cardiovascular disorders (44), and blood pressure (45). It has been suggested that the decreasing incidence of dementia could reflect a delay in dementia onset (8,46). Because more people now survive into high ages, a delay in dementia onset could result in a higher incidence in this age group. The results of our study indicate that this might not be the case, or that onset might be delayed into even higher ages.

The decline in prevalence and incidence of dementia was mainly found among women, which was reflected by a diminishing sex difference in later-born cohorts. Thus, we observed no sex difference at age 85, but a higher prevalence in women compared to men at age 88 among those born 1901–02 and 1923–24, a sex difference which had disappeared in those born 1930. However, the number of men was small, giving low statistical power. A potentially larger decline among women is interesting as the prevalence of dementia is most often reported to be higher among women compared to men in this age group (19,47). Some studies also report that women have a higher incidence of dementia than men after the age of 85–90 years (48,49), except (50). The reason behind the higher prevalence and incidence of dementia among female octogenarians is not clear. However, a longer life expectancy among women (51), a survival effect among men, or more prolonged exposure to estrogens (52) may play a role. We observed no within-cohort sex differences at age 85, but a higher prevalence in women compared to men at age 88 among those born 1901–02 and 1923–24 (yet not among those born 1930). The sex difference in dementia prevalence may be moving toward higher ages in later-born cohorts. Our finding that the decline in prevalence and incidence of dementia was more accentuated among women is in line with a Swedish study showing that a decrease in the incidence up to age 89 years was mainly driven by women (15). This was further supported by the US Framingham study conducted between 1977 and 2013 in a population aged above 65 (8). In contrast, the rural study from northern Sweden conducted between 1995–98 and 2001–03 (12) and the Spanish study conducted between 1988–89 and 1994–96, reported a more pronounced decline in the prevalence among men among those younger than 85 (38). In addition, the MRC-CFAS study from the United Kingdom reported a decline in dementia incidence between 1989–94 and 2008–11 in both sexes aged 65 and older (9). However, in individuals aged 85 and older, they showed a decrease only among men. The disparate findings might also be due to the low number of individuals above age 85 in the studies.

Our finding that the incidence and prevalence of dementia declined more in women than in men is similar to several other findings on time trends in mixed ages of older people. Thus, we have previously reported a more accentuated decline among women regarding several risk factors for dementia, for example, sleep problems (42), neuroticism and depression (43), obesity, overweight and cardiovascular disorders (44), and blood pressure (45). Others have reported that women have gained more healthy years (53) and improved more in cognitive function (54) than men. It has also been suggested that women have gained higher educational levels across our study period, which may have contributed to a reduced sex difference in cognitive performance. This suggests that the secular changes in access to education among women might attenuate the differences in risk for dementia (22,55). These larger improvements in health and risk factors among women compared to men could partly explain why the decrease in dementia incidence and prevalence was mainly observed among women.

We found a high specificity and a low sensitivity of IPR or CDREG for dementia diagnoses, which is in line with previous studies (20). Thus, only 44% of identified cases in the examination received a diagnosis when combining registers, and 42% when using only the IPR. The level of sensitivity corresponds to the previous work from Sweden showing sensitivity for dementia diagnosis in this age group of 47% (95% CI: 42–52) (20), despite that we used the Inpatient registry only, whereas the previous work included both the Inpatient and Outpatient registers. The reason why we only used the IPR is that the Outpatient registry was not included in the National Register until 2001 (56), making comparisons with the first cohort impossible. Had we been able to use the Outpatient registry as well, the sensitivity would likely be higher. It has also been shown that the IPR gives a later age of diagnosis than diagnosis by study examinations (20). This may lead to a lower incidence using register data in this high age group with increased mortality and lower sensitivity in the IPR (20). One problem with using register data when studying time trends is that these also reflect changes in diagnostic criteria and awareness of dementia in health care systems and in the population, potentially leading to a change in care-seeking behavior. In line with this, studies investigating time trends in dementia using register data have found a steep increase up to the early 2010s, however, followed by stabilization or even a decrease (31,32). Also, an Australian study investigating the prevalence of dementia in individuals who accessed aged care showed a decline between 2008 and 2014 (57).

We had expected that the register would detect more cases in recent cohorts than 3 decades ago due to increased awareness of dementia in society. However, the sensitivity of the registers remained stable over time. Because the sensitivity of register data is low, the incidence among those lost to follow-up is likely underestimated.

Among the study’s strengths are the 3 large samples of 85–90-year olds examined with identical methods and diagnostic criteria over 3 decades. We also need to discuss some possible limitations and methodological considerations. First, in some subgroups, the sample size is small, especially in men, which may result in low statistical power. Second, the response rate ranges from 60% to 63% in the 3 cohorts, which is considered good. Still, we cannot exclude the possibility that the response rate at baseline among those with dementia or that characteristics of nonresponders have changed between cohorts. Also, in the follow-ups, the drop-out due to death decreased between cohorts (21%, 17%, and 13% in cohorts 1901–02, 1923–24, and 1930, respectively), and the response rate among survivors increased (68.9%, 73.4%, and 82.4%). With a higher response rate and more survivors in the later-born cohorts, dementia cases were more likely to be detected. This might have underestimated the decrease in dementia incidence. Third, we used registry data from the IPR and CDREG to detect dementia in drop-outs. Approaches to account for dementia cases among drop-outs vary between studies, which may influence incidence rates. These include, for example, using likelihood models (9), information from close informants and medical practitioners (10), and informants only in persons with low cognition at baseline and for all drop-outs information from the regional institute for outpatient mental health care (6).

In conclusion, the prevalence and incidence of dementia decreased substantially over 3 decades among octogenarians. This might slow down the projected increase in cases of dementia expected by the increasing number of octogenarians during the following decades.

## Supplementary Material

glad071_suppl_Supplementary_Material

## Data Availability

All data and analyses generated during the current study are available from the corresponding author on reasonable request.
