# Supplementary material for: Decreasing Incidence and Prevalence of Dementia Among Octogenarians: A Population-Based Study on 3 Cohorts Born 30 Years Apart
Source: J Gerontol A Biol Sci Med Sci. 2023 Feb 27;78(6):1069–77. doi: 10.1093/gerona/glad071 (PMC10235204; doi:10.1093/gerona/glad071)
Supplement: glad071_suppl_Supplementary_Material [file glad071_suppl_supplementary_material.pdf]

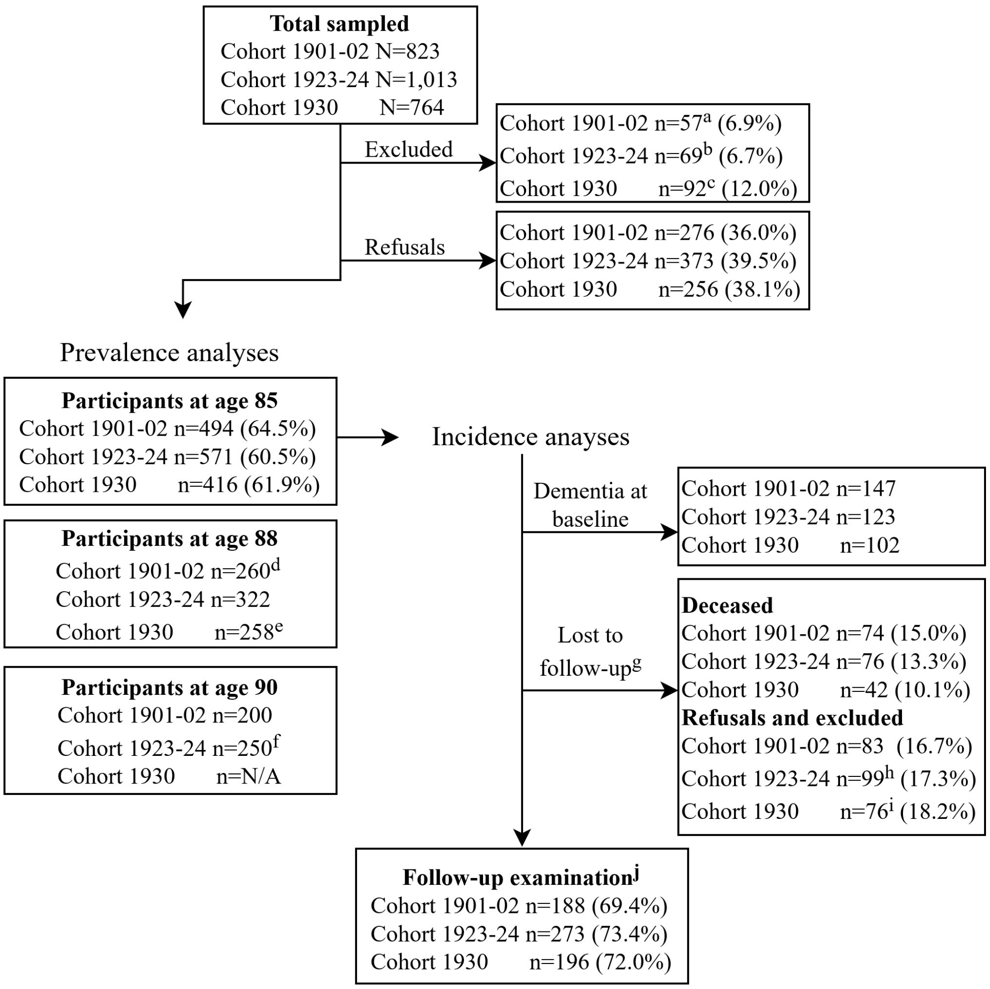

Supplementary Fig. 1 Sample flowchart

Figure legend

N/A = Not applicable, cohort 1930 was not examined at age 90 due to the Covid-19 pandemic.

[a] 43 died before examination, 14 moved/ not traceable

[b] 40 died before examination, 10 moved/ not traceable, 19 did not speak Swedish

[c] 42 died before examination, 6 moved/ not traceable, 31 did not speak Swedish, 13 technical issue

[d] including 12 individuals that did not participate at baseline

[e] including 34 individuals that did not participate at baseline

[f] including 41 individuals that did not participate at baseline

[g] Deceased, refusals and excluded. Followed only by register data

[h] 94 refusals, 5 excluded due to language

[i] 73 refusals, 1 excluded due to no contact, 1 due to language, and 1 due to emigrated

[j] Response rate among survivors

Table s1. Dementia prevalence at ages 85, 88 and 90

	Total % (n/N)	Women % (n/N)	Men % (n/N)	Sex and education standardization	Sex standardized %	Education standardization %
Age 85						
1901-02	29.8 (147/494)	30.8 (108/351)	27.3 (39/143)	22.3	29.4	22.1
1923-24	21.5 (123/571)	23.7 (85/359)	17.9 (38/212)	21.0	21.4	21.1
1930	24.5 (102/416)	23.9 (60/251)	25.5 (42/165)	24.3	24.5	24.8
Age 88						
1901-02	41.9 (109/260)*	46.4 (89/192)	29.4 (20/68)	32.8	39.6	33.6
1923-24	28.0 (90/322)[a]	31.7 (65/205)	21.4 (25/117)	27.4	27.6	27.4
1930	21.7 (55/254)	22.4 (36/161)	20.4 (19/93)	22.0	21.6	21.6
Age 90						
1901-02	41.5 (83/200)	44.1 (63/143)	35.1 (20/57)	38.1	40.5	38.5
1923-24	37.2 (93/250)	36.9 (59/160)	37.8 (34/90)	36.9	37.2	36.8
1930	N/A	N/A	N/A	N/A	N/A	N/A

Note. Standardized rates based on 2021 Swedish census data. N/A = Not applicable, cohort 1930 was not examined at age 90 due to the Covid-19 pandemic.

[a] $p<0.05$ for sex difference within cohort. See supplementary Table 2 for details

Table s2. Within cohort comparisons between men and women of prevalence of dementia at age 85, 88 and 90

	Men<br>% (n/N)	Women<br>% (n/N)	OR (95% CI)	p-value
Age 85				
1901-02	27.3 (39/143)	30.8 (108/351)	1.19 (0.77-1.83)	0.441
1923-24	17.9 (38/212)	23.7 (85/359)	1.42 (0.93-2.18)	0.107
1930	25.5 (42/165)	23.9 (60/251)	0.92 (0.58-1.45)	0.719
Age 88				
1901-02	29.4 (20/68)	46.4 (89/192)	2.07 (1.15-3.76)	0.016
1923-24	21.4 (25/117)	31.7 (65/205)	1.71 (1.01-2.91)	0.048
1930	20.4 (19/93)	22.4 (36/161)	1.12 (0.60-2.10)	0.719
Age 90				
1901-02	35.1 (20/57)	44.1 (63/143)	1.46 (0.77-2.75)	0.246
1923-24	37.8 (34/90)	36.9 (59/160)	0.96 (0.56-1.64)	0.887
1930	N/A	N/A	N/A	N/A

Note. OR = odds ratio derived from logistic regressions. CI = confidence intervals. N/A = Not applicable as cohort 1930 were not examined at age 90 due to the Covid-19 pandemic

Table s3. Cumulative incidence of dementia between age 85 and 89

	Total	Women	Men	Sex and education standardization	Sex standardized	Education standardization
Dementia cases/ N						
1901-01	47[a]/347	40/243	7/104			
1923-24	46[b]/448	32/274	14/174			
1930	19[c]/314	11/191	8/123			
Person years						
1901-01	964	676	288			
1923-24	1214	746	468			
1930	845	517	328			
Incidence rate (95% CI)						
1901-01	48.8[d] (36.6-64.9)	59.2 (43.4-80.7)	24.3 (11.6-51.0)	43.4	38.3	46.5
1923-24	37.9 (28.4-50.6)	42.9 (30.3-60.7)	29.9 (17.7-50.5)	39.7	35.1	37.7
1930	22.5 (14.3-35.3)	21.3 (11.8-38.4)	24.4 (12.2-48.8)	24.2	23.1	22.9

Note. Incidence rate per 1,000 person-years. Standardized rates based on 2021 Swedish census data.

[a] 44 identified at follow-up examination, another 3 by register only

[b] 42 identified at follow-up examination, another 4 by register only

[c] 15 identified at follow-up examination, another 4 by register only

[d] $p<0.05$ for sex difference within cohort. See supplementary Table s4 for details.

Table s4. Within cohort comparisons between men and women of incidence of dementia between age 85 and 89

	Men				Women				
	No	Person years	Dementia cases	IR (95% CI)	No	Person years	Dementia cases	IR (95% CI)	IRR (95% CI)
1901-02	104	288.1	7	24.3 (11.6-51.0)	243	676.1	40	59.2 (43.4-80.7)	2.43 (1.09-5.43)
1923-24	174	467.8	14	29.9 (17.7-50.5)	274	746.2	32	42.9 (30.3-60.7)	1.43 (0.76-2.69)
1930	123	328.2	8	24.4 (12.2-48.8)	191	516.6	11	21.3 (11.8-38.4)	0.87 (0.35-2.17)

Note. IR = Incidence rate. IRR = incidence rate ratio derived from Poisson regressions. CI = confidence intervals

Table s5. Sensitivity and specificity of the IPR and CDREG in octogenarians

	National InPatient Register or Cause of death register				National InPatient Register or Cause of death register			
	No of cases	True positive (n)	Sensitivity[a]	95% CI	No of NON-cases	True negative (n)	Specificity[a]	95% CI
Total	492	216	43.9	39.5-48.4	1031	1014	98.4	97.4-99.0
1901-02	198	84	42.4	35.4-49.6	308	305	99.0	97.2-99.8
1923-24	165	73	44.2	36.5-52.2	406	398	98.0	96.2-99.1
1930	129	59	45.7	36.9-54.7	317	311	98.1	95.9-99.3

Note.

[a] Diagnoses in the IPR or CDREG up to age 89

No of cases = Total number of cases identified at examination at age 85 or 88

True positive = Cases in the IPR or CDREG also identified at the examination

No of NON-cases = Total number of participants without dementia at examination

non-case = Total number of participants without dementia both in the IPR or CDREG and at the examinations

Table s6. Sensitivity and specificity of the IPR

	National InPatient Register				National InPatient Register			
	No of cases	True positive (n)	Sensitivity[a]	95% CI	No of NON-cases	True negative (n)	Specificity[a]	95% CI
Total	492	216	41.9	37.5-46.4	1031	1018	98.7	97.9-99.3
1901-02	198	84	42.4	35.4-49.6	308	305	99.0	97.2-99.8
1923-24	165	73	40.6	33.0-48.5	406	400	98.5	96.8-99.5
1930	129	59	42.6	34.0-51.6	317	313	98.7	96.8-99.7

Note.

[a] Diagnoses in the IPR up to age 89

No of cases = Total number of cases identified at examination at age 85 or 88

True positive = Cases in the IPR also identified at the examination

No of NON-cases = Total number of participants without dementia at examination

non-case = Total number of participants without dementia both in the IPR and at the examinations
